# Supplementary material for: Exploring the lived experience of families with a COVID-19 positive child: The journey from a critical grounded theory approach
Source: PLoS One. 2023 Mar 1;18(3):e0282481. doi: 10.1371/journal.pone.0282481 (PMC9977002; doi:10.1371/journal.pone.0282481)
Supplement: Attachment — Submitted filename: PONE-D-22-15338 Response to reviewers .docx [file pone.0282481.s001.docx]

Response to reviewers

Manuscript Number: PONE-D-22-15338

We thank the reviewers for their thoughtful feedback and outline our responses to their comments below.

1) The authors intended to investigate the experiences within a family with a child who has a SARS-CoV-2 PCR positive test result. Even though the manuscript is well written, I couldn't find an added value to the literature. Most of the study's findings are already known and they fail to fulfill the real world's practical needs. Finally, the take-home message of the study was not clear.

**Author response:** This study is the first to our knowledge to identify the disconnect between the lived experiences of families with young children and early pandemic government messaging and policies designed for the general public. As our participants expressed, inadequate consideration of children and families in public health messaging and policy contributed to psychological distress and stigmatisation. The critical realist analysis approach, in particular, is a unique feature of this study. Using this lens, we not only identified unmet needs of families at specific timepoints, we also developed specific recommended strategies to improve their experience and inform future pandemic planning (Table 2).

We have highlighted the take-home message of the manuscript in the abstract and include the following text in the conclusion (p24):

“This study highlights the importance of family-centred messaging and policies during the ongoing COVID-19 pandemic and in future pandemics or disease outbreaks. Families have unique experiences and support needs that may not be addressed by the systems and communication directed to the general public.”

2) page 12 line 110 - text reading "that is, theoretical sampling", please add "within the sample". I suggest this be added to prevent confusion with the statement above that theoretical sampling was not possible.

**Author response:** We agree that this may be confusing so have amended the text in line 115 to remove the second reference to theoretical sampling.

3) Table 1 - in two rows "Dad" is underlined. Is there a meaning to the underlining. If so, please provide a key. If not please remove the underlining.

**Author response:** The underlining was in error and has been removed.

4) page 13 - text reading "Some participants were concerned about being identified in the media, since employers and schools of positive cases were sometimes reported" - do you mean "sometimes notified"? I assume so given the context, or do you mean that employers and schools were reported to public health authorities, or reported in the media? Please clarify.

**Author response:** We have clarified that parents and children who tested positive, particularly if they were the index case, were concerned about being identified publicly. The media reported details about the location of positive cases identified in schools or workplaces and the cases were often able to be identified in small outbreaks, causing distress for families. The amended sentence reads “Some participants were concerned about being identified publicly as employers and schools of positive cases were often reported in the media.” (lines 282-283, p 13)

5) page 21 line 21 - Do you think that the feelings of stigma would have been grounded in the low incidence of COVID-19 within Australia at that time (particularly relative to other countries)? Do you think that there would be less stigma now that COVID-19 is more prevalent within Australia? A discussion about the stigma of low-prevalence diseases and acceptance of high-prevalence diseases might make a valuable contribution, particularly in the context of the stigma associated with the new monkey-pox 'epidemic'.

**Author response:** We appreciate the reviewer’s thoughtful suggestion. We agree the issue around stigma for COVID-19 positive cases, especially index children who triggered a school closure, was intense at the start of the pandemic and of great concern to many parents. This has not been well reported in the literature, especially from Australia.

COVID-19 related stigma was reported around the world in the early part of the pandemic, suggesting that it was driven by confusion, fear and lack of therapeutics or vaccines at that time, more than by familiarity with the disease. However, it is possible that the limited case numbers in Australia and the intense restrictions associated with individual cases and their movements (e.g. postcode-wide lockdowns, school closures) exacerbated stigma. We have expanded our discussion and included additional literature related to the issue of stigma (lines 456-468, pp21-22).

6) page 23 line 25 - word missing "due those under age 5 being" should read "due to those ..."

**Author response:** This has been amended.

# JOURNAL REQUIREMENTS

1.  Please ensure that your manuscript meets PLOS ONE's style requirements, including those for file naming. The PLOS ONE style templates can be found at

<https://journals.plos.org/plosone/s/file?id=wjVg/PLOSOne_formatting_sample_main_body.pdf>  and

<https://journals.plos.org/plosone/s/file?id=ba62/PLOSOne_formatting_sample_title_authors_affiliations.pdf>

**Author response:** We have amended the manuscript and file names as requested.

2. We note that you have indicated that data from this study are available upon request. PLOS only allows data to be available upon request if there are legal or ethical restrictions on sharing data publicly. For more information on unacceptable data access restrictions, please see http://journals.plos.org/plosone/s/data-availability#loc-unacceptable-data-access-restrictions.

In your revised cover letter, please address the following prompts:

a) If there are ethical or legal restrictions on sharing a de-identified data set, please explain them in detail (e.g., data contain potentially sensitive information, data are owned by a third-party organization, etc.) and who has imposed them (e.g., an ethics committee). Please also provide contact information for a data access committee, ethics committee, or other institutional body to which data requests may be sent

b) If there are no restrictions, please upload the minimal anonymized data set necessary to replicate your study findings as either Supporting Information files or to a stable, public repository and provide us with the relevant URLs, DOIs, or accession numbers. For a list of acceptable repositories, please see http://journals.plos.org/plosone/s/data-availability#loc-recommended-repositories.

We will update your Data Availability statement on your behalf to reflect the information you provide.

**Author response:** As per the study protocol approved by the Royal Children’s Hospital Human Research Ethics Committee, de-identified participant data will be made available on request for use by future researchers from a recognised research institution whose proposed use of the data has been ethically reviewed and approved by an independent committee. Contact details: +61 3 9345 5044 or email [rch.ethics@rch.org.au](mailto:rch.ethics@rch.org.au)

3. Please include your full ethics statement in the ‘Methods’ section of your manuscript file. In your statement, please include the full name of the IRB or ethics committee who approved or waived your study, as well as whether or not you obtained informed written or verbal consent. If consent was waived for your study, please include this information in your statement as well.

**Author response:** We have included the full ethics details and noted that written informed consent was obtained.
