# Supplementary material for: Exploring the lived experience of families with a COVID-19 positive child: The journey from a critical grounded theory approach
Source: PLoS One. 2023 Mar 1;18(3):e0282481. doi: 10.1371/journal.pone.0282481 (PMC9977002; doi:10.1371/journal.pone.0282481)
Supplement: Attachment — Submitted filename: PONE-D-22-15338 Response to reviewers 2.docx [file pone.0282481.s002.docx]

Response to reviewers #2

Manuscript Number: PONE-D-22-15338

We thank the editor for their feedback on this manuscript. Our responses are outlined below.

1) In lines 79-80: authors have revised the manuscript to include obtaining a "written informed consent". However, the authors claimed that this was done over the phone by the research assistants. Authors should explain and provide further information on how they obtained written consent.

**Author response:** Using the REDCap eConsent functionality, we were able to collect written informed consent electronically (i.e., participants could provide a digital signature to the consent form). We have added clarification around this point in lines 80-81.

2) In lines 95-96: How did you assure the rigor and trustworthiness of the online data collection, i.e. using Zoom? To guarantee procedural clarity, I would consider adding information or a reference to the approach you followed.

**Author response:** We appreciate this suggestion. Our interview procedures were designed to address the main potential challenges of Zoom interviewing (e.g. lack of rapport, unstable internet issues, privacy). We have described this briefly and included a reference that informed our approach.

3) In lines 73-174: Readers may be perplexed by the term "boogas" in the quotations because it is a slang term. This problem could be solved by using a generally recognized phrase in brackets after the word.

**Author response:** Thank you for identifying this issue. We have defined the term as ‘mucous’ in the quote (line 175).

4) I acknowledge that the two children's interviews were insufficient to undertake data analysis; nevertheless, given the journey component of the proposed framework, is there any possibility that one or both of the children interviewed as positive cases may be connected in the article as an illustrative case? This could be linked in the results - this would call policymakers' and academics' attention to the fact that, while your study was unable to catch more children, the stories and narratives of these children could improve the primary takeaways and criticality of the data.

**Author response:**  We appreciate this suggestion, but unfortunately the two children mentioned did not complete full interviews. They were both young and offered only a handful of short responses or elaborations when prompted by their parents. We did attempt to recruit families with older children who could participate more fully in the interviews, but unfortunately we were not successful with this effort. Future research focusing specifically on the views of children and adolescents would be very valuable, given the reports of mental health challenges experienced by children during this period.
